# Combining in vitro protein detection and in vivo antibody detection identifies potential vaccine targets against *Staphylococcus aureus* during osteomyelitis

**DOI:** 10.1007/s00430-016-0476-8

**Published:** 2016-09-14

**Authors:** P. Martijn den Reijer, Marjan Sandker, Susan V. Snijders, Mehri Tavakol, Antoni P. A. Hendrickx, Willem J. B. van Wamel

**Affiliations:** 1 Department of Medical Microbiology and Infectious Diseases, Erasmus Medical Center, s-Gravendijkwal 230, 3015 CE Rotterdam, The Netherlands; 2 Department of Orthopaedics, Erasmus Medical Center, Rotterdam, The Netherlands; 3 Department of Medical Microbiology, University Medical Center Utrecht, Utrecht, The Netherlands

**Keywords:** *Staphylococcus aureus*, Osteomyelitis, Biofilm, Virulence factors, Luminex

## Abstract

Currently, little is known about the in vivo human immune response against *Staphylococcus aureus* during a biofilm-associated infection, such as osteomyelitis, and how this relates to protein production in biofilms in vitro. Therefore, we characterized IgG responses in 10 patients with chronic osteomyelitis against 50 proteins of *S. aureus*, analyzed the presence of these proteins in biofilms of the infecting isolates on polystyrene (PS) and human bone in vitro, and explored the relation between in vivo and in vitro data. IgG levels against 15 different proteins were significantly increased in patients compared to healthy controls. Using a novel competitive Luminex-based assay, eight of these proteins [alpha toxin, *Staphylococcus aureus* formyl peptide receptor-like 1 inhibitor (FlipR), glucosaminidase, iron-responsive surface determinants A and H, the putative ABC transporter SACOL0688, staphylococcal complement inhibitor (SCIN), and serine–aspartate repeat-containing protein E **(**SdrE)] were also detected in a majority of the infecting isolates during biofilm formation in vitro. However, 4 other proteins were detected in only a minority of isolates in vitro while, vice versa, 7 proteins were detected in multiple isolates in vitro but not associated with significantly increased IgG levels in patients. Detection of proteins was largely confirmed using a transcriptomic approach. Our data provide further insights into potential therapeutic targets, such as for vaccination, to reduce *S. aureus* virulence and biofilm formation. At the same time, our data suggest that either in vitro or immunological in vivo data alone should be interpreted cautiously and that combined studies are necessary to identify potential targets.

**Electronic supplementary material:**

The online version of this article (doi:10.1007/s00430-016-0476-8) contains supplementary material, which is available to authorized users.

## Introduction

*Staphylococcus aureus* is the most common causative organism of osteomyelitis [1–3], which is defined as an infection of the bone and is associated with significant morbidity [1, 4, 5]. Treatment is often difficult and requires surgery in addition to antibiotics [5]. Osteomyelitis is associated with the formation of bacterial biofilms [3, 6], which are defined as complex communities of bacteria enclosed in a polymer matrix that differ significantly in their gene expression and protein production compared to free-living bacteria [7]. Biofilm formation is believed to increase resistance against antibiotics and the host immune system, which further complicates the treatment of such infections [3, 8, 9].

Together with the increasing incidence of resistant *S. aureus* isolates [10, 11], the difficult treatment of osteomyelitis underscores the need for alternative treatment strategies. One such strategy is the development of a vaccine [12]. However, so far no clinically successful vaccine against *S. aureus* has been developed, despite the promising results of vaccines targeting diverse virulence factors of this pathogen in animal models [13–15], including an animal model for osteomyelitis [16]. Possibly, so far clinically evaluated vaccines might have failed because these were based on single antigens, while the awareness is currently increasing that multiple virulence factors of *S. aureus* should be targeted to undermine bacterial virulence [12, 13].

Although diverse virulence factors of *S. aureus* have been associated with biofilm formation [17–21], so far only a limited number of studies has focussed on the presence of multiple virulence factors simultaneously in biofilms [22, 23]. In addition, most of these studies used in vitro and/or animal models, while it remains unclear whether proteins expressed in these models are also involved in infection in humans. Finally, the immunogenic potential of proteins that are specifically expressed during a biofilm-associated infection remains unknown, as studies examining the immune response against *S. aureus* in patients have so far mainly focused on acute infections, such as bacteremia [24–29].

The aims of the present study were to characterize IgG responses in 10 patients with chronic osteomyelitis against 50 proteins of *S. aureus*, to analyze the presence of these proteins in biofilms of *S. aureus* isolates from the same patients on polystyrene (PS) and human bone in vitro, and to explore the relation between in vivo and in vitro data. All 50 proteins are functionally well-characterized virulence factors which have been a major focus of many immunoproteomic studies, including vaccination trails, in both animal models [13–15] and humans [25, 29–31]. This study provides further insights into the presence and immunogenicity of these proteins during a biofilm-associated chronic infection in humans.

## Materials and methods

### Human serum and tissue

All patient serum used in this study was obtained from coded leftover material from routine diagnostic blood samples as described previously [29]. This procedure was approved, and the acquisition of additional written consent was waived specifically for this study by the Medical Ethics Committee of the Erasmus University Medical Center Rotterdam (MEC-2007-106). Human bone was derived from surplus tissue obtained during routine orthopedic surgical procedures, and all tissue was directly anonymized upon arrival at the laboratory. This procedure was approved, and the acquisition of additional written consent was waived by the Medical Ethics Committee of the Erasmus University Medical Center Rotterdam (MEC-2004-322). None of the authors were involved in the direct collection of either blood or tissue from patients. Only qualified physicians (MdR and MS) had access to potentially identifying patient information.

### Patients

Ten patients diagnosed with chronic osteomyelitis at the Erasmus Medical Center between March 2007 and March 2011 were included in this study (all male, median age 62 years, interquartile range (IQR) 49–69 years). Chronic osteomyelitis was defined as a history of at least 1 year of clinical and radiological signs indicative of osteomyelitis combined with the isolation of *S. aureus* from at least one deep bone culture. Isolates from 9 patients could be retrieved for this study. The cause of osteomyelitis varied, being iatrogenic following surgery (4 patients), fracture or other trauma (3 patients), diabetic ulcer (2 patients), or hematologic metastasis from another focus (1 patient). Infections were localized in the femur (3 patients), small bones of the foot (3 patients), tibia (2 patients), ulna and sternum (each 1 patient). Nasal carrier status for *S. aureus* was not tested.

A median number of 4 serum samples were collected per patient (IQR 2–5) over a median period of 26 days following the most recent positive bone culture (IQR 9.5–85.5 days). Peak antibody levels of each patient were compared with those measured in single serum samples of 10 previously described bacteremia patients (80 % male, median age 64 years, IQR 45–84) and 20 previously described, age-matched controls (all male, median age 62 years, IQR 57–67) who had no record of a clinically apparent infection in at least 6 months [29]. *S. aureus* nasal carrier status was tested neither in patients nor in controls.

### Bacterial strains and genotyping

*S. aureus* isolates obtained from deep bone cultures were identified on the basis of colony and microscopic morphology and Slidex Staph Plus agglutination testing (bioMérieux, Marcy l’Etoile, France). Identification was confirmed by *spa*-PCR, and all isolates were *spa*-typed [32]. All isolates were methicillin-sensitive as determined by cefoxitin disk diffusion according to the CLSI criteria [33]. Antimicrobial susceptibility to additional antibiotics was determined using the VITEK^®^ 2 system with card AST-P549 (bioMérieux).

Isolates were further typed using pulsed-field gel electrophoresis (PFGE) with *Sma1*-digested chromosomal DNA as described previously [34]. Relatedness among the PFGE profiles was evaluated using Bionumerics software (version 3.0; Applied Maths, Ghent, Belgium). Finally, all isolates were screened with PCR for the presence of 50 genes using previously described primers [29].

### Preparation of human serum and measurement of antibodies

Serum samples were collected in BD Vacutainer^®^ SST II Advance plastic serum tubes, which were centrifuged for 3 min at 1680*g* and stored at 4 °C. For long-term use, serum was aliquoted in 1.5-ml Eppendorf^®^ tubes and stored at −80 °C.

IgG levels against 50 recombinant *S. aureus* proteins were measured in serum samples using a bead-based flow cytometry technique (xMAP^®^; Luminex^®^ Corporation, Austin, TX, USA), as previously described [29]. Protein names are described in Online Resource 1. Proteins were coupled to xMAP^®^ carboxylated beads (Luminex Corporation) as described previously [29, 35]. All measurements were taken in duplicate, and the median fluorescence intensities (MFIs), a semiquantitative measure of antibody levels, were averaged. Duplicate measurements for which the coefficient of variation was larger than 25 % were excluded from further analysis. All measurements were corrected for non-specific background signal by subtracting the MFIs of control beads not coupled to any protein.

### Preparation of human bone

Fresh human bone was obtained in the operating room from patients receiving a total hip prosthesis, for purposes other than this study, in the Erasmus Medical Center from the period of January 2012 until February 2013. All operations were performed by the same orthopedic surgeon (Dr. P.K. Bos). After removing the femoral head, a small portion of the surplus tissue was cut using a saw and directly transported to the laboratory in sterile saline. Spongious bone was harvested from the tissue and cut into small pieces fitting in a 96-well plate. The samples were rinsed repeatedly with saline until all blood was visibly removed.

### Biofilm formation on polystyrene and human bone

A routine biofilm model was used as described before [20, 36–38]. Briefly, overnight cultures of *S. aureus* grown on sheep blood agar were suspended in Iscove’s Modified Dulbecco’s Medium (IMDM, Life technologies, Carlsbad, CA, USA) without phenol red until an OD660 of 2.0 was reached. One µl of this bacterial suspension was added to 199 µl of IMDM in sterile, 96-well polystyrene plates (Greiner Bio-one GmbH, Kremsmuenster, Austria). Duplicate wells not inoculated with bacteria served as sterile controls. Plates were then incubated at 37 °C and gentle shaking at 200 rpm for various intervals. Biofilm mass was measured by staining with 1 % crystal violet. OD was measured at 490 nm.

Alternatively, standard-sized pieces of freshly isolated human bone were washed in sterile water and then added to 199 µl of IMDM in the same 96-well plates in which biofilms on PS were grown. One µl of the same bacterial suspension as described above was added before incubation. Duplicate wells with bone tissue were not inoculated to serve as sterile controls.

### Multiplex bead assay for assessment of protein levels in biofilms

A previously described multiplex competition Luminex^®^ assay (CLA) was used to indirectly detect the presence of 50 IgG-accessible proteins in biofilms [39, 40]. In brief, biofilms grown on PS and bone were washed once with ice-cold PBS supplemented with 0.5 % (wt/v) sodium azide (Sigma-Aldrich) at 1, 8, 24, or 48 h to remove non-adherent bacteria. Bone tissue with attached biofilms was transferred to a clean well. Biofilms were then incubated for 35 min at 8 °C and continuous shaking (500 rpm) with 200 µl of a 1:200 dilution of polyclonal human IgG (PHG), isolated using the HiTrap™ Protein G HP column according to the manufacturer’s guidelines (GE Healthcare Bio-sciences, Piscataway, New Jersey, USA), from pooled serum of 40 healthy volunteers [41].

After incubation, the remaining non-bound IgG antibody levels in recovered PHG samples were measured using the multiplex bead-based flow cytometry technique (xMAP^®^, Luminex corporation), with recombinant proteins covalently coupled to the beads, as described above. As negative controls PHG samples incubated with empty PS wells or sterile bone pieces were included in all experiments.

Next, the percentage decrease in the levels of specific IgG antibodies for each protein was calculated in relation to the negative control. The percentage decrease can be considered as a semiquantitative measure of the protein-specific antibody absorption from PHG by the biofilm, thus indirectly reflecting the presence of the particular *S. aureus* protein in the biofilm [39, 40]. The average percentage decrease plus two times the standard deviation, obtained at 24- and 48-h biofilm growth, for two non-*S. aureus* control proteins [*Streptococcus pneumoniae* putative proteinase maturation protein A (PpmA) and human metapneumovirus surface protein (hMPV)] and all *S. aureus* proteins of which genes were not present in an isolate were chosen as cutoff value (35 % at 24-h biofilm growth and 42 % at 48 h, respectively).

### Reverse transcriptase PCR

Biofilms were grown on PS for 8 and 24 h in 96-well plates in 200 µl of IMDM. Biofilms harvested from 8 wells were washed in PBS, resuspended, pooled, and centrifuged at 4000 rpm for 10 min at 4 °C. Pellets were resuspended in 200 µl of RNA protect™ Bacterial reagents (Qiagen), stabilized for 5 min, and then centrifuged for 10 min at 4 °C. The pellet was dissolved in 1 ml of RNA-pro solution (Fast RNA Pro Blue kit, MP Biomedicals) and stored at −20 °C until use. RNA was isolated using the Fast RNA Pro Blue kit according to the manufacturer’s protocol. Each 10 µg of isolated RNA was treated twice with 2 U TURBO DNase (Ambion, Life Technologies). The reaction was stopped by adding 0.2 volumes of DNase inactivation reagent (Ambion) and incubated for 2 min at ambient temperature. RNA-containing supernatants were collected by centrifugation (1.5 min at 9000*g* at ambient temperature), and each 2 µg DNase-treated RNA was treated with 2 U DNase I (Fermentas, Fisher Scientific). One µg of prepared RNA was transcribed into cDNA using 200 U RevertAid H Minus Reverse transcriptase (Fermentas), 4 µl of 5 × reaction buffer (Fermentas), 20 U of RiboLock RNase inhibitor (Fermentas), and 2 µl of 10 mM dNTP mix (Fermentas) in a final volume of 20 µl of DEPC-treated water. This was incubated for 60 min at 42 °C and then terminated by heating at 70 °C for 5 min. For each RNA sample, a negative control without reverse transcriptase was processed similarly. The presence of cDNA in all samples was examined using PCR as described previously [29].

### Cryo-scanning electron microscopy

The clinical isolate from one osteomyelitis patient was allowed to form biofilms on human bone for 24 h as described above. Next, *S. aureus* in bone was fixed for 15 min with 1 % (v/v) glutaraldehyde (Sigma) in phosphate-buffered saline (PBS) at room temperature. Samples were washed twice with PBS to remove excess fixative and were subsequently serially dehydrated by consecutive incubations in 1 ml of 25 % (v/v) and 50 % (v/v) ethanol–PBS, 75 % (v/v) and 90 % (v/v) ethanol–H_2_O, and 100 % ethanol (2x), followed by 50 % ethanol–hexamethyldisilazane (HMDS) and 100 % HMDS (Sigma) and air-dried overnight at room temperature. After overnight evaporation of HMDS, bone samples were mounted on 12-mm specimen stubs (12 mm, Agar Scientific) and coated with gold to 1 nm using a Quorum Q150R sputter coater at 20 mA prior to examination with a Phenom PRO Table-top scanning electron microscope (PhenomWorld).

### Statistics

Mean IgG levels between patient groups and controls were compared using one-way ANOVA. All data were logarithmically transformed to obtain equal variances between groups, checked with Levene’s test for equality of variances. For proteins that were associated with a significant difference in the ANOVA, additional least significant difference (LSD) post hoc tests were performed for further group comparisons.

Correlation between biofilm mass and the percentage decrease in specific IgG levels, as obtained with CLA, was determined by calculating the nonparametric Spearman’s rank correlation coefficient (r_s_).

In all cases, *p* values ≤ 0.05 were considered as statistically significant. IBM^®^ SPSS^®^ Statistics version 21 (IBM corporation, Armonk, NY, USA) was used for statistical analysis. Graphics were made using GraphPad Prism version 5 (GraphPad Inc., La Jolla, CA, USA).

## Results

### Genetic typing of clinical *S. aureus* isolates from osteomyelitis patients

**Fig. 1:**
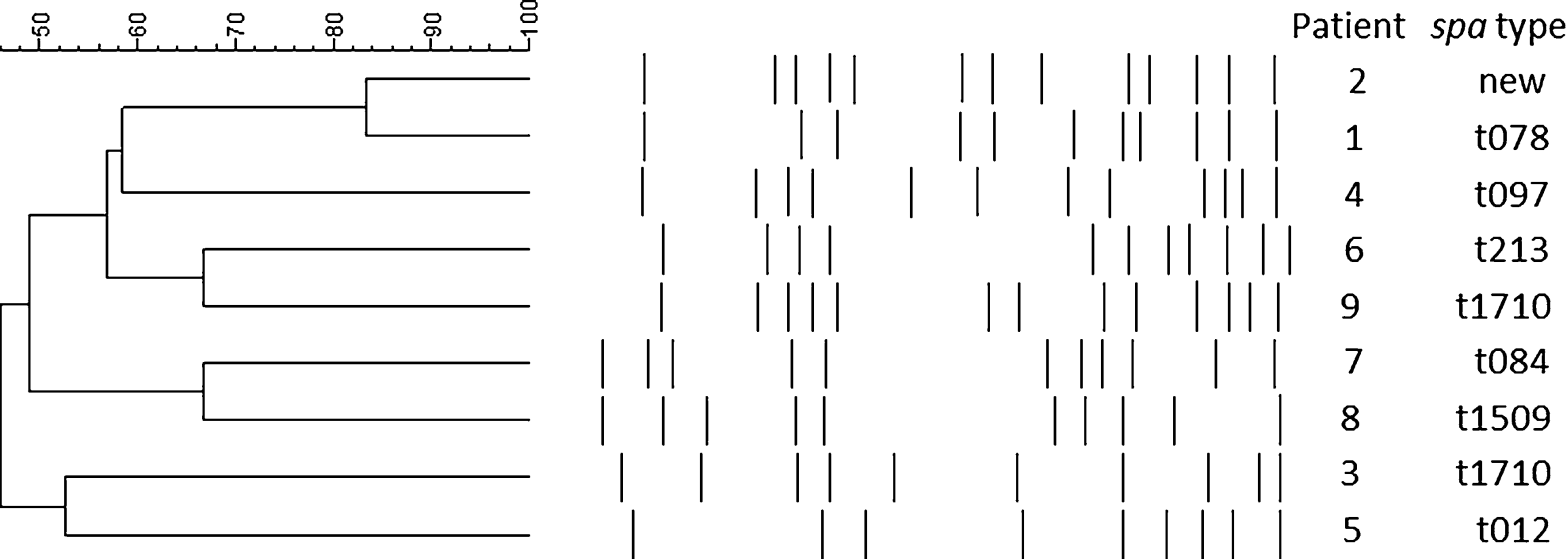
Genotypes of 9 clinical isolates from bone cultures. Pulsed-field gel electrophoresis data and *spa* types are shown. An isolate of the tenth patient described in this study could not be retrieved

Ten patients who were diagnosed with chronic osteomyelitis caused by *S. aureus*, confirmed by deep bone culture, were included in this study. *S. aureus* isolates could be retrieved from bone cultures of 9 patients, and these were genotyped using *spa* typing and PFGE analysis. Eight out of 9 isolates contained different *spa* types, and PFGE analysis revealed an overall lack of relatedness between isolates (Fig. 1).

In addition to genetic typing, the presence of genes encoding 50 virulence factors of *S. aureus* was examined in the 9 clinical isolates plus the extensively studied reference strain NCTC 8325 using PCR (Table 1, Table S1 in Online Resource 2). Notably, the genes *eta* and *etb*, *lukS* and *lukF*, *ssl9*, *Tst* and the genes encoding 13 enterotoxins were only detected in a minority of the 10 isolates. The other 31 genes were present in at least half or more of all isolates.

**Table 1:** Presence of genes, mRNA, and protein during biofilm formation by 10 *S. aureus* isolates

Protein^a^	Gene	Functional class	Strains with gene present^b^	Biofilms on polystyrene		Biofilms on polystyrene			Biofilms on human bone		
				mRNA present^b^		Significant reduction in specific IgG^b^			Significant reduction in specific IgG^b^		
				8 h	24 h	8 h	24 h	48 h	8 h	24 h	48 h
Alpha toxin	*hla*	Toxin	10	10	10	5	7	9	6	6	8
CHIPS	*chps*	Immune modulator	7	4	4	3	5	7	6	5	6
ClfA	*clfA*	Surface protein	10	4	4	3	6	10	7	9	10
ClfB	*clfB*	Surface protein	10	10	10	2	7	9	5	8	8
FlipR	*Flr*	Immune modulator	9	7	5	0	4	5	3	5	5
FnbA	*fnbA*	Surface protein	10	10	10	–	2	5	1	5	6
Glucosaminidase	*Atl*	Housekeeping	10	10	10	10	10	10	10	10	10
IsaA	*isaA*	Housekeeping	10	10	10	10	10	10	10	10	10
IsdA	*isdA*	Surface protein	9	9	9	9	9	9	4	8	9
Nuc	*nuc*	Housekeeping/toxin	8	5	1	7	7	8	7	8	8
SACOL0688	*MntC*	Housekeeping	10	10	10	9	10	10	10	10	10
SCIN	*scn*	Immune modulator	10	9	8	9	9	9	9	9	9
IsdH	*isdH*	Surface protein	10	10	10	3	3	6	2	2	1
Lipase	*lip*	Housekeeping/toxin	10	10	10	1	6	8	1	2	1
SasG	*sasG*	Surface protein	7	6	6	1	1	1	7	4	2
SdrD	*sdrD*	Surface protein	7	5	5	1	1	3	2	6	6
SdrE	*sdrE*	Surface protein	7	7	6	1	1	3	5	5	5

^a^Only proteins are shown that were found in at least half of 9 clinical isolates and strain NCTC8325 with both transcriptomic analysis and CLA at minimally one time point on minimally one surface. From top to bottom, protein groups are shown that were detected in a majority of isolates on both PS and bone, mostly on PS, or mostly on human bone, respectively

^b^Number of isolates in which, respectively, the gene, mRNA, or protein was detected are shown. – indicates that the gene, mRNA, or protein was not detected in any isolate

### Biofilm formation by clinical *S. aureus* isolates on polystyrene and human bone

**Fig. 2:**
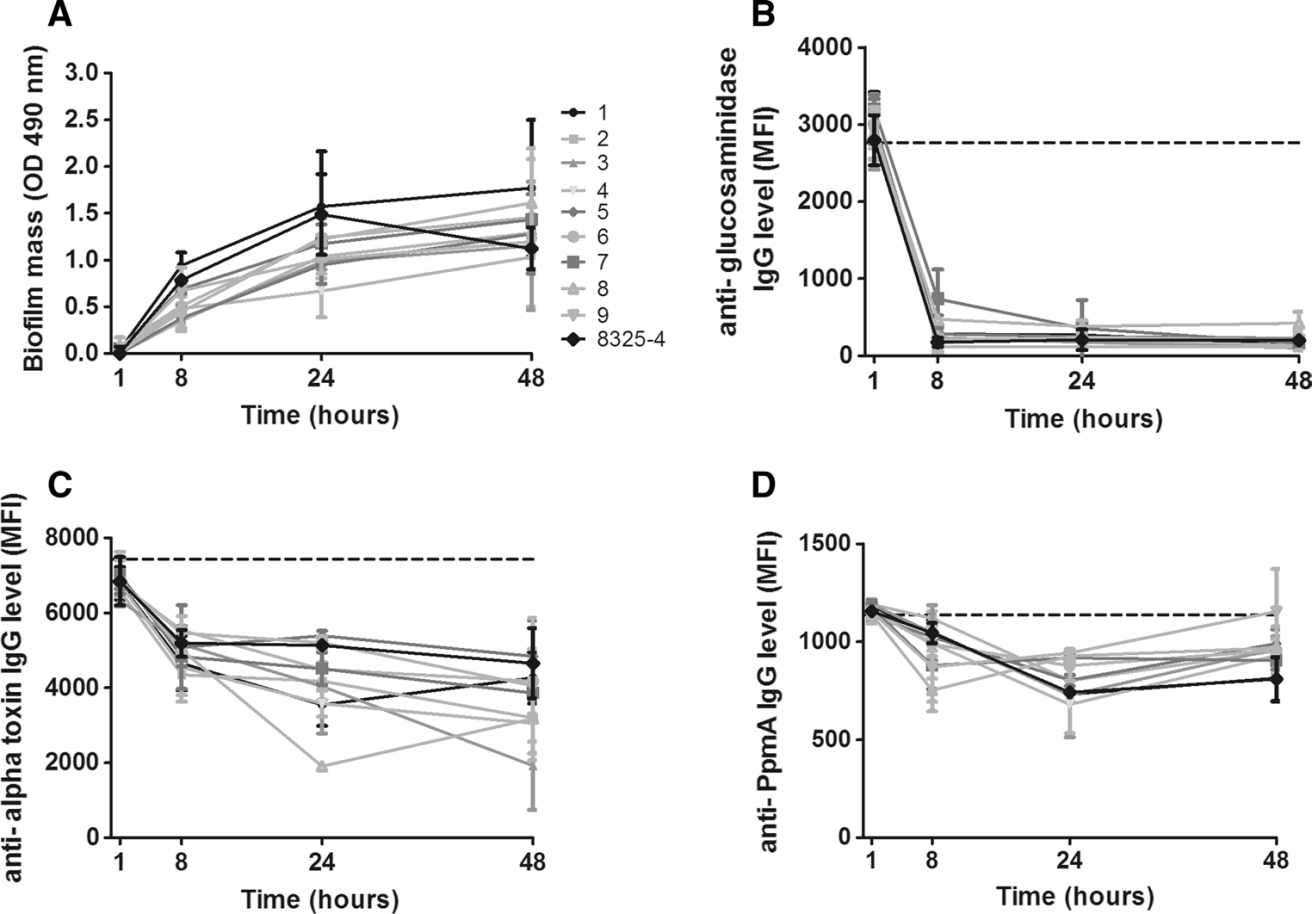
Detection of *S. aureus* proteins during biofilm formation on PS. Biofilm mass formed on PS by 9 clinical isolates and reference strain NCTC 8325 (**a**). The levels of remaining non-bound IgG directed against specific proteins in PHG after incubation with the biofilms [expressed as mean fluorescence intensity (MFI)], indirectly and inversely reflecting protein presence, are shown for glucosaminidase (**b**), alpha toxin (**c**), the non-*S. aureus* control protein PpmA (**d**). Please note the difference in range of MFI on the *y*-axis between Fig b–d. *Dashed horizontal lines* indicate the average MFI of sterile controls. *Symbols* and *error bars* indicate mean and SD of two separate experiments

**Fig. 3:**
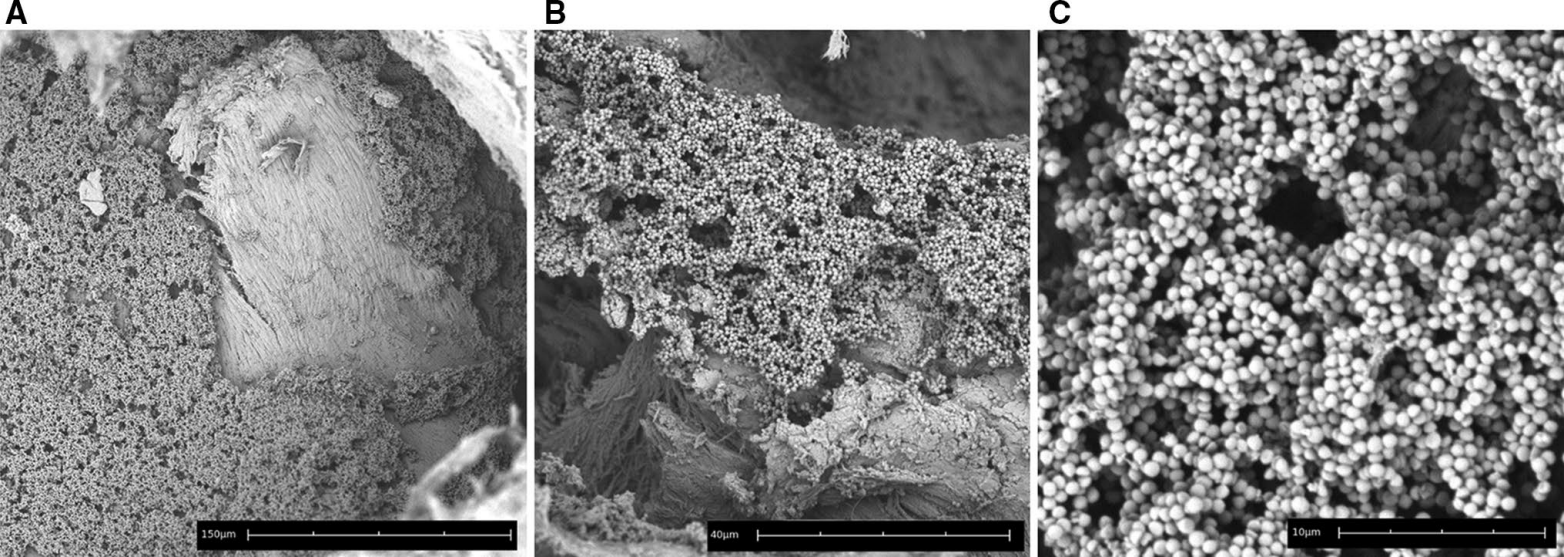
Biofilm formation of *S. aureus* on human bone. Cryo-scanning electron microscopy of one *S. aureus* isolate after 24 h of biofilm formation on human bone. Please note the different magnifications indicated by the *scale bars*, respectively, being 150 μm (**a**), 40 μm (**b**), and 10 μm (**c**)

Next, the ability of the 9 clinical isolates and the reference strain NCTC 8325 to form biofilms on polystyrene (PS) and freshly isolated human bone were examined. Using crystal violet staining, we confirmed that all isolates were able to stably form biofilms on PS, although the amount of biofilm mass varied (Fig. 2a). Furthermore, using scanning electron microscopy, layers of tightly adherent bacteria resembling biofilms on human bone after 24-h incubation were visualized for one clinical isolate (Fig. 3a–c). These results indicate the development of mature biofilms on both surfaces.

### Characterization of IgG antibody response against *S. aureus* proteins in osteomyelitis patients

Total IgG levels directed against 50 proteins of *S. aureus* were prospectively measured during variable intervals within the study period (median of 26 days, interquartile range 9.5–85.5 days) in serum samples of the 10 patients (median of 4 samples per patient, interquartile range 2–5). Measurements for efb, EsxB, PrsA, and SA0486 were excluded from further analysis due to low signal intensities with coefficients of variation larger than 25 % between duplicate experiments.

In line with previous data [24, 26, 28], the height of all protein-specific IgG levels was heterogeneous between patients (Table 2, Table S2 in Online Resource 2), without any clear pattern in the course over time (Fig. S1 in Online Resource 3). IgG levels remained detectable up to 250 days within the study period. Comparison of IgG levels between the osteomyelitis patients, 10 patients suffering from a *S. aureus* bacteremia and 20 age-matched non-infected controls, revealed that mean IgG levels differed significantly for 15 out of 46 proteins between groups (ANOVA *p* < 0.05, Table 2). Mean IgG levels against 10 proteins [*Staphylococcus aureus* formyl peptide receptor-like 1 inhibitor (FLIPr), glucosaminidase, gamma-hemolysin B, iron-responsive surface determinant A (IsdA), leukocidins D and F, SACOL0699, staphylococcal complement inhibitor (SCIN), and Staphylococcal superantigen-like proteins 3 and 5 (SSL 3 and 5)] were significantly higher in both osteomyelitis and bacteremia patients compared to controls, IgG levels against 4 proteins (alpha toxin, exfoliative toxin A (ETA), serine–aspartate repeat-containing protein E **(**SdrE), and enterotoxin M) were significantly higher only in osteomyelitis patients, and IgG levels against 1 protein (IsdH) were only higher in bacteremia patients (Table 2). None of the mean protein-specific IgG levels differed significantly between osteomyelitis and bacteremia patients. Notably, IgG levels against all proteins were readily detectable in the control group, and IgG levels against the remaining proteins that are not mentioned above did not differ significantly between patients and controls (Table S2, Fig. S2).

**Table 2:** Proteins of *S. aureus* associated with significantly increased IgG levels in patients

Protein^a^	Gene	Function class	Mean IgG level control patients (±SD; *N* = 20)	Mean IgG level bacteremia patients (±SD; *N* = 10)^b^	Mean IgG level osteomyelitis patients (±SD; *N* = 10)^b^	*P* value ANOVA^c^	*P* value Post hoc analysis^d^
FlipR	*flr*	Immune modulator	1864 (±1569)	4490 (±3485)	3656 (±2143)	0.007	0.019
Glucosaminidase	*Atl*	Housekeeping	5273 (±2827)	86,780 (±3612)	8377 (±3882)	0.005	0.019
HlgB	*hlgB*	Toxin	6328 (±4290)	10,838 (±2924)	10,917 (±3627)	0.002	0.007
IsdA	*isdA*	Surface protein	3722 (±4532)	6534 (±3993)	5016 (±2756)	0.006	0.023
LukD	*lukD*	Toxin	6311 (±3988)	9512 (±3514)	9369 (±3581)	0.024	0.044
LukF	*lukF*	Toxin	1105 (±880)	1851 (±772)	2025 (±917)	0.001	0.005
SACOL0688	*MntC*	Housekeeping	839 (±650)	3849 (±4290)	2572 (±2432)	0.001	0.005
SCIN	*scn*	Immune modulator	3665 (±3322)	7939 (±3805)	7545 (±3782)	<0.000	0.002
SSL3	*ssl3*	Immune modulator	4679 (±3068)	8186 (±4746)	6955 (±3673)	0.011	0.042
SSL5	*ssl5*	Immune modulator	1929 (±1307)	4675 (±3315)	3932 (±2827)	0.001	0.014
Alpha toxin	*hla*	Toxin	8895 (±4419)	11,610,9 (±5117)	14,884,3 (±3749)	0.037	0.011
ETA	*eta*	Toxin	893 (±1425)	1238 (±1946)	2874 (±4178)	0.026	0.007
SdrE	*sdrE*	Surface protein	293 (±298)	399 (±385)	651 (±441)	0.026	0.007
SEM	*sem*	Toxin	526 (±542)	1155 (±1595)	1322 (±966)	0.028	0.011
IsdH	*isdH*	Surface protein	825 (±867)	2579 (±3130)	2732 (±4407)	0.034	0.011

^a^Only proteins are shown that were associated with significantly increased IgG levels in at least one patient group. From top to bottom, protein groups are shown that were associated with significantly increased IgG levels in both bacteraemia and osteomyelitis patients, only in osteomyelitis patients, and only in bacteraemia patients compared to controls, respectively

^b^Only the peak IgG levels of patients were included for comparison

^c^ *P* value of ANOVA test comparing all three patient groups. *P* values <0.05 were considered as significant

^d^Groups were additionally compared using least significant difference (LSD) post hoc tests. The smallest *p* values, related to the significantly differing groups, are shown

### Detection of proteins of *S. aureus* isolates during biofilm formation on PS

We used a competitive Luminex-based assay (CLA) [39, 40] to establish the presence of the same 50 proteins during biofilm formation by 9 of the infecting isolates described above. In line with previous results [39], biofilm mass-dependent absorption of specific IgG for several *S aureus* proteins such as glucosaminidase (Fig. 2b) was detected, while no such reduction was seen for IgG specific against non-*S. aureus* control proteins such as the putative protease maturation protein A (PpmA) of *Streptococcus pneumoniae* (Fig. 2d). Similar to the differences in biofilm mass (Fig. 2a), the reduction in IgG levels against most proteins, such as alpha toxin, was heterogeneous between isolates (Fig. 2c). The average amount of formed biofilm mass and the average percentage reduction in IgG levels correlated significantly for all proteins (e.g., alpha toxin: r_s_ −0.77, *p* < 0.0001). Based on the percentage decrease in IgG levels against the non-*S. aureus* control proteins and against all *S. aureus* proteins of which the gene was not found in an isolate, cutoff values for protein detection of at least 35 % decrease in specific IgG at 24-h and 42 % at 48-h biofilm formation were calculated. CLA measurements for four proteins (ESX-1-associated factor B (EsxB), extracellular fibrinogen-binding protein (efb), foldase-protein PrsA, and the putative protein SA0486) were excluded from further analysis due to low MFIs with standard deviations larger than 25 % between repeated CLA measurements.

For the 31 genes that were found in at least half of the 10 isolates, 14 proteins were detected in the majority of gene-positive isolates at minimally one time point (8, 24, and/or 48 h) during biofilm formation on PS (Table 1): the surface proteins clumping factor A and B (ClfA and B), fibronectin-binding protein A (FnbpA), IsdA and H, the housekeeping proteins glucosaminidase, immunodominant antigen A (IsaA), lipase, nuclease, and the putative ABC transporter SACOL0688, the immune modulators FlipR, SCIN, and chemotaxis inhibitory protein of *S. aureus* (CHIPS), and alpha toxin.

For the genes that were found in a minority of isolates, the proteins exfoliative toxin A (ETA) and the staphylococcal enterotoxins A, B, D, Q, and R were detected in one to maximally three gene-positive isolates during biofilm formation on PS (Table S1 in Online Resource 2). Using the above-mentioned cutoff values, no proteins were detected when the corresponding gene was absent in an isolate.

The lack of detection of several secreted proteins, despite the presence of corresponding genes in isolates (Table S1 in Online Resource 2**)**, prompted us to repeat CLA experiments using the medium covering biofilms instead of biofilms self. Results obtained with medium showed similar reduction in IgG levels against all proteins, including secreted proteins such as alpha toxin, compared to results obtained with biofilms (Fig. S3 in Online Resource 3). This suggests that the lack of protein detection is not due to false-negative signals. CLA data were further confirmed by the detection of specific mRNA at 8 and/or 24 h of biofilm formation for all detected proteins (Table 1; Table S1 in Online Resource 2).

### Detection of proteins of *S. aureus* isolates during biofilm formation on human bone

**Fig. 4:**
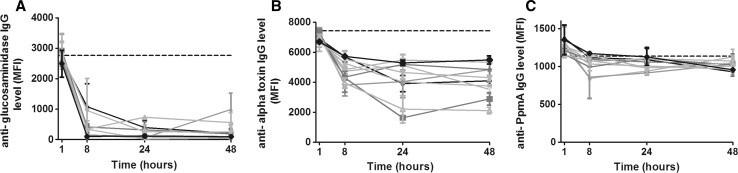
Detection of *S. aureus* proteins during biofilm formation on human bone. The levels of remaining non-bound IgG directed against specific proteins after incubation of PHG with biofilms of 9 clinical isolates and strain NCTC 8325 on bone (expressed in MFI) are shown for glucosaminidase (**a**), alpha toxin (**b**), and the non-*S. aureus* control protein PpmA (**c**). *Dashed horizontal lines* indicate the average MFI of sterile controls. *Symbols* and *error bars* indicate mean and SD of two separate experiments

In an attempt to examine the expression of *S. aureus* virulence factors in an environment that more closely resembles the in vivo conditions during osteomyelitis, we repeated the above-mentioned experiments with biofilms grown on human bone. Generally, the same patterns of reduction in specific IgG levels were observed as for biofilms on PS, including for glucosaminidase, alpha toxin, and the PpmA control protein (Fig. 4a–c). Compared to biofilm formation on PS, similar percentages of non-specific reduction in IgG levels were found, prompting us to use the same cutoff values.

For the 31 genes that were found in half or more of the 10 isolates, 15 proteins were detected in the majority of gene-positive isolates at minimally one time point (8, 24, and/or 48 h) during biofilm formation on human bone (Table 1). These were largely the same proteins that were also detected during biofilm formation on PS, with the exception of IsdH and lipase, which were detected in only a minority of isolates at all time points. On the other hand, *S. aureus* surface protein G (SasG) and SD-repeat-containing proteins D and E (SdrD and E) were detected in most isolates during biofilm formation on bone but not on PS at several time points (Table 1).

Notably, for the genes that were found in a minority of isolates, all Staphylococcal enterotoxins were detected in 1–4 gene-positive isolates during biofilm formation on bone, which were more isolates compared to the results obtained with biofilms on PS (Table S1 in Online Resource 2).

### Comparison of in vivo antibody responses with in vitro protein detection

For the 31 genes that were found in at least half of the 10 isolates, 8 *S. aureus* proteins were associated with significantly increased IgG levels in patients and detectable in the majority of the gene-positive isolates during biofilm formation on PS and/or bone: alpha toxin, FlipR, glucosaminidase, IsdA and H, SACOL0688, SCIN, and SdrE. Among the less common genes, enterotoxin M was associated with significantly increased IgG levels in patients, and it was detectable in multiple isolates specifically during biofilm formation on bone.

In contrast to the above-mentioned results, the toxins hemolysin gamma-B and leucocidin D and the immune modulators SSL3 and 5 were associated with significantly increased IgG levels in patients but detected in only a minority of gene-positive isolates during biofilm formation in vitro. Vice versa, the surface proteins ClfA and B and the housekeeping proteins IsaA, lipase, and nuclease were detected in a majority of strains during biofilm formation in vitro but were not associated with significantly increased IgG levels in patients. The latter was also true for the less common enterotoxins B and Q, for which genes were present in 3 and 2 isolates, respectively.

## Discussion

In this study, we characterized the IgG response against 50 functionally diverse proteins of *S. aureus* in patients with chronic osteomyelitis. IgG levels against 14 diverse virulence factors, such as alpha toxin, the surface protein IsdA, and the housekeeping protein glucosaminidase, were significantly increased in osteomyelitis patients compared to healthy controls. Interestingly, a comparison with peak IgG levels of patients suffering from a *S. aureus* bacteremia revealed little difference, suggesting that immunological exposure to specific virulence factors is similar during both infections. Indeed, bacteria residing within a biofilm can dislodge and potentially cause a bacteremia [42]. This implicates that it might be difficult to find serological markers that can specifically discriminate between a *S. aureus* osteomyelitis and other sorts of infection. Moreover, in the context of identifying potential targets for example vaccination, this implicates that results of immunological in vivo studies should be interpreted cautiously when searching for targets that are specifically involved in biofilm formation.

To gain further insight into the involvement of the 50 proteins mentioned above in biofilm formation, we screened for the presence of these proteins during biofilm formation of 9 clinical isolates and reference strain NCTC-8325 on polystyrene (PS) and freshly isolated human bone, using a novel competitive Luminex-based assay (CLA). In general, we observed a clear variation in the reduction in protein-specific IgG levels between isolates, which can largely be explained by differences in the amount of biofilm mass formed by each isolate. This variability in biofilm mass is in line with previous results, which might be related to the different genetic backgrounds of the isolates [43].

For the 31 proteins of which genes were present in at least half of the 9 clinical isolates and NCTC 8325, 12 proteins were detected in at least half of all isolates at minimally one time point (8, 24, and/or 48 h) during biofilm formation on both PS and bone. Several of these proteins, especially surface proteins such as ClfB and FnbpA, already have a known role in biofilm formation [44–46]. In addition, several detected proteins, including IsaA, SACOL0688, and glucosaminidase, have been successfully evaluated as vaccine targets in animal infection models, some specifically in an osteomyelitis model [16, 47]. Our results further indicate the potential use of these proteins as vaccine targets by demonstrating their actual production during biofilm formation by genetically diverse clinical isolates ex vivo and, based on antibody responses, also during osteomyelitis in human patients in vivo.

Although the CLA results between biofilm formation on PS and bone generally agreed well, detection of a few proteins differed. Lipase and IsdH were mostly detected on PS while the biofilm-associated surface proteins SasG, SdrD, and SdrE were mostly detected on bone. In addition, also the genetically less common enterotoxins were detected relatively more often on bone. The presence of SdrE and D specifically during biofilm formation on bone might be explained by bacterial attachment to the calcium-rich extracellular matrix, as these proteins are structurally dependable on calcium [48]. Although we cannot currently explain the other observed differences, we have demonstrated before using CLA that different circumstances can impact protein detection in vitro and ex vivo [39, 40]. In this context, production of some of the above-mentioned proteins, including diverse enterotoxins, is regulated by the accessory gene regulatory (Agr) quorum-sensing system [49, 50]. Possibly, this system is activated differently upon interaction of *S. aureus* with either PS or bone. Lastly, next to culture circumstances also the timing of measurement can be influential, as demonstrated by the detection of SasG mostly during early biofilm formation (8 h), which is in line with its established role during the early accumulation phase [51]. Together, these findings implicate that results from in vitro models should be interpreted cautiously, and both timing and circumstances during the measurement should be taken into account.

Combination of in vitro and in vivo results suggests that 8 proteins, of which most are already discussed above, are both immunogenic in patients and are detectable in the majority of clinical isolates during biofilm formation. At the same time, some toxins and immune modulators were associated with significantly increased IgG levels in patients but not detected during biofilm formation in vitro. Possibly, our in vitro biofilm model might not adequately reflect the in vivo situation in patients, or the measured IgG levels might be mounted against proteins produced by planktonic growing bacteria within the patient. Moreover, IgG levels against the leukocidins might be false-positive due to the presence of cross-reactive antibodies, which has been established before in our assay [29]. In contrast to the above-mentioned proteins, other proteins such as the ubiquitously present clumping factors and IsaA were detected in vitro but not associated with significantly increased IgG levels in patients. This might well be explained by the high IgG levels that we found in both healthy controls and in patients, thereby eliminating any statistically significant difference between groups. Indeed, antibody titers to diverse *S. aureus* proteins appear to be ubiquitously present in the general population [24, 28], and this appears to be mostly independent of nasal carrier ship for *S. aureus* [41] cautiously.

A limitation of the current Luminex assay is that its sensitivity is potentially influenced by amino acid sequence diversity, which is described for multiple proteins described in this study [52–55]. However, in general sequence diversity has been described within certain limits, and these differences do not significantly impact our assay sensitivity, based on cross-reactivity within our assay as shown earlier for the leukocidin components and hemolysin gamma-B [29] and also the more variable, different isotype forms of the FnBPA A domain [52] (data in submission). In addition, we only compare average IgG levels for each protein separately, further levelling out potential variation in assay sensitivity due to protein diversity. Taken together, we are confident that the current assay allows us to screen for the presence of specific antibodies and proteins [29, 40].

The 50 proteins included in the current assay were chosen based on their established roles in *S. aureus* pathogenesis and the corresponding host immune response. However, there are many more, e.g. cytosol-based proteins of *S. aureus* that remain less-characterized in this context, yet could also be potentially interesting therapeutic targets. We believe that these proteins will be an interesting addition to future immunoproteomic studies, although this is not the focus of the current study.

We conclude that functionally diverse virulence factors of *S. aureus* are present during biofilm formation by genetically diverse isolates on PS and human bone in vitro, and that some of these proteins are immunogenic in vivo. These observations merit more mechanistic studies to elucidate the function of specific proteins and the regulation of their expression during *S. aureus* biofilm formation. However, the present data further suggest that multiple proteins, such as the ubiquitously present and immunogenic IsdA or SA0688, could be potential targets for novel agents such as a multivalent vaccine to prevent or treat biofilm-associated infections in patients. Combined studies using both in vitro models and immunological assays in patients in vivo can help in identifying novel therapeutic targets.

## Electronic supplementary material

Below is the link to the electronic supplementary material.

Supplementary material 1 (DOCX 12 kb)

Supplementary material 2 (DOCX 23 kb)

Supplementary material 3 (DOC 209 kb)
