# Supplementary material for: Combining in vitro protein detection and in vivo antibody detection identifies potential vaccine targets against Staphylococcus aureus during osteomyelitis
Source: Med Microbiol Immunol. 2016 Sep 14;206(1):11–22. doi: 10.1007/s00430-016-0476-8 (PMC5263195; doi:10.1007/s00430-016-0476-8)
Supplement: Supplementary file 1 — Supplementary material 1 (DOCX 12 kb) [file 430_2016_476_MOESM1_ESM.docx]

**Online resource 1. Bacterial proteins used for the Luminex assay**

All *S. aureus* proteins used for Luminex experiments were 6x His-tagged recombinant proteins. The following proteins were coupled to xMAP® beads (Luminex Corporation): protein secretion system ESX-1-associated factor B (EsxB); Nuclease (Nuc); peptidoglycan hydrolase (LytM); immunodominant antigen A (IsaA); glucosaminidase; lipase; foldase-protein PrsA; clumping factor A and B (ClfA and ClfB); SD-repeat containing proteins D and E (SdrD and SdrE); iron-responsive surface determinants A and H (IsdA and IsdH); fibronectin-binding proteins A and B (FnbpA and FnbpB); extracellular fibrinogen-binding protein (Efb); *S. aureus* surface protein G (SasG); staphylococcal complement inhibitor (SCIN); chemotaxis inhibitory protein of *S. aureus* (CHIPS); formyl peptide receptor-like inhibitory protein (FLIPr); alpha toxin; gamma-hemolysin B (HlgB); leukocidins D, E, F and S (LukD, LukE, LukF and LukS); staphylococcal enterotoxins A-E, G-I, M-O, Q, R (SEA–SEE, SEG-SEI, SEM-SEO, SEQ, SER); exfoliative toxins A and B (ETA and ETB); toxic shock syndrome toxin 1 (TSST-1); staphylococcal superantigen-like proteins 1 (SSL1), SSL-3, -5, -9, -10 and -11 and hypothetical proteins SA0486 and SA0688. The following purified non-staphylococcal proteins were also coupled to xMAP beads as negative controls: *Moraxella catarrhalis* IgD-binding protein (MID); *Streptococcus pneumoniae* putative proteinase maturation protein A (PpmA) and human metapneumovirus surface protein (hMPV).
