# Supplementary material for: Combining in vitro protein detection and in vivo antibody detection identifies potential vaccine targets against Staphylococcus aureus during osteomyelitis
Source: Med Microbiol Immunol. 2016 Sep 14;206(1):11–22. doi: 10.1007/s00430-016-0476-8 (PMC5263195; doi:10.1007/s00430-016-0476-8)
Supplement: Supplementary file 2 — Supplementary material 2 (DOCX 23 kb) [file 430_2016_476_MOESM2_ESM.docx]

**Online Resource 2. Supplementary Tables.**

**Table S1. Detection of genes, mRNA and proteins during biofilm formation of 10 S. aureus strains on PS and human bone.**

|  |  |  |  | Biofilms on polystyrene | |  | Biofilms on polystyrene | | |  | Biofilms on human bone | | |
| --- | --- | --- | --- | --- | --- | --- | --- | --- | --- | --- | --- | --- | --- |
| Protein | Gene | Strains with gene present^1^ |  | Strains with mRNA present^1^ | |  | Strains with significant reduction in specific IgG^1^ | | |  | Strains with significant reduction in specific IgG^1^ | | |
|  |  |  |  | 8 hrs | 24 hrs |  | 8 hrs | 24 hrs | 48 hrs |  | 8 hrs | 24 hrs | 48 hrs |
| Alpha toxin | *hla* | 10 |  | 10 | 10 |  | 5 | 7 | 9 |  | 6 | 6 | 8 |
| CHIPS | *chps* | 7 |  | 4 | 4 |  | 3 | 5 | 7 |  | 6 | 5 | 6 |
| ClfA | *clfA* | 10 |  | 4 | 4 |  | 3 | 6 | 10 |  | 7 | 9 | 10 |
| ClfB | *clfB* | 10 |  | 10 | 10 |  | 2 | 7 | 9 |  | 5 | 8 | 8 |
| Efb | *efb* | 10 |  | 10 | 10 |  | *ND* | *ND* | *ND* |  | *ND* | *ND* | *ND* |
| EsxB | *esxB* | 10 |  | 5 | 5 |  | *ND* | *ND* | *ND* |  | *ND* | *ND* | *ND* |
| ETA | *eta* | 1 |  | - | - |  | - | - | 1 |  | - | 1 | 1 |
| ETB | *etb* | - |  | *ND* | *ND* |  | *ND* | *ND* | *ND* |  | *ND* | *ND* | *ND* |
| FlipR | *flr* | 9 |  | 7 | 5 |  | 0 | 4 | 5 |  | 3 | 5 | 5 |
| FnbA | *fnbA* | 10 |  | 10 | 10 |  | - | 2 | 5 |  | 1 | 5 | 6 |
| FnbB | *fnbB* | 5 |  | 5 | 5 |  | 1 | 3 | 2 |  | - | 2 | 1 |
| Glucosaminidase | *Atl* | 10 |  | 10 | 10 |  | 10 | 10 | 10 |  | 10 | 10 | 10 |
| HlgB | *hlgB* | 9 |  | 9 | 9 |  | - | - | - |  | - | 1 | - |
| IsaA | *isaA* | 10 |  | 10 | 10 |  | 10 | 10 | 10 |  | 10 | 10 | 10 |
| IsdA | *isdA* | 9 |  | 9 | 9 |  | 9 | 9 | 9 |  | 4 | 8 | 9 |
| IsdH | *isdH* | 10 |  | 10 | 10 |  | 3 | 3 | 6 |  | 2 | 2 | 1 |
| Lipase | *lip* | 10 |  | 10 | 10 |  | 1 | 6 | 8 |  | 1 | 2 | 1 |
| LukD | *lukD* | 8 |  | 8 | 8 |  | 0 | 2 | 4 |  | 1 | 3 | 4 |
| LukE | *lukE* | 8 |  | 8 | 8 |  | 0 | 2 | 3 |  | 0 | 1 | 1 |
| LukF | *lukF* | - |  | *ND* | *ND* |  | *ND* | *ND* | *ND* |  | *ND* | *ND* | *ND* |
| LukS | *lukS* | - |  | *ND* | *ND* |  | *ND* | *ND* | *ND* |  | *ND* | *ND* | *ND* |
| LytM | *lytM* | 10 |  | 10 | 10 |  | 1 | 1 | 4 |  | 1 | 4 | 2 |
| Nuc | *nuc* | 8 |  | 5 | 1 |  | 7 | 7 | 8 |  | 7 | 8 | 8 |
| PrsA | *prsA* | 10 |  | 10 | 10 |  | *ND* | *ND* | *ND* |  | *ND* | *ND* | *ND* |
| SACOL0486 | *sacol0486* | 6 |  | 5 | 5 |  | *ND* | *ND* | *ND* |  | *ND* | *ND* | *ND* |
| SACOL0688 | *MntC* | 10 |  | 10 | 10 |  | 9 | 10 | 10 |  | 10 | 10 | 10 |
| SasG | *sasG* | 7 |  | 6 | 6 |  | 1 | 1 | 1 |  | 7 | 4 | 2 |
| SCIN | *scn* | 10 |  | 9 | 8 |  | 9 | 9 | 9 |  | 9 | 9 | 9 |
| SdrD | *sdrD* | 7 |  | 5 | 5 |  | 1 | 1 | 3 |  | 2 | 6 | 6 |
| SdrE | *sdrE* | 7 |  | 7 | 6 |  | 1 | 1 | 3 |  | 5 | 5 | 5 |
| SEA | *sea* | 2 |  | 2 | 2 |  | 0 | 0 | 2 |  | 2 | 2 | 2 |
| SEB | *seb* | 3 |  | 3 | 3 |  | 1 | 2 | 3 |  | 3 | 3 | 3 |
| SEC | *sec* | - |  | *ND* | *ND* |  | *ND* | *ND* | *ND* |  | *ND* | *ND* | *ND* |
| SED | *sed* | 1 |  | 1 | 1 |  | 1 | 1 | 1 |  | 1 | 1 | 1 |
| SEE | *see* | - |  | *ND* | *ND* |  | *ND* | *ND* | *ND* |  | *ND* | *ND* | *ND* |
| SEG | *seg* | 3 |  | 3 | 3 |  | - | - | - |  | 1 | 2 | 1 |
| SEH | *seh* | 1 |  | 1 | 1 |  | - | - | - |  | 1 | 1 | 1 |
| SEI | *sei* | 4 |  | 3 | 3 |  | - | - | - |  | 3 | 3 | 3 |
| SEM | *sem* | 4 |  | 4 | 3 |  | - | - | - |  | 4 | 2 | 3 |
| SEN | *sen* | 4 |  | 4 | 4 |  | - | - | - |  | 1 | 1 | 1 |
| SEO | *seo* | 4 |  | 4 | 4 |  | - | - | - |  | - | 1 | - |
| SEQ | *seq* | 2 |  | 2 | 2 |  | 1 | 2 | 2 |  | 2 | 2 | 2 |
| SER | *ser* | 1 |  | 1 | 1 |  | 0 | 1 | 1 |  | 1 | 1 | 1 |
| SSL1 | *ssl1* | 10 |  | 9 | 8 |  | - | - | 1 |  | - | 1 | 1 |
| SSL3 | *ssl3* | 10 |  | 10 | 9 |  | - | - | 1 |  | - | 1 | 1 |
| SSL5 | *ssl5* | 6 |  | 5 | 6 |  | - | - | - |  | - | 1 | - |
| SSL9 | *ssl9* | 2 |  | - | - |  | - | - | - |  | - | - | - |
| SSL10 | *ssl10* | 5 |  | 1 | 2 |  | - | - | - |  | - | 1 | 1 |
| SSL11 | *ssl11* | 5 |  | 1 | 2 |  | 1 | 1 | 2 |  | - | - | - |
| TSST1 | *Tst* | - |  | *ND* | *ND* |  | *ND* | *ND* | *ND* |  | *ND* | *ND* | *ND* |

^12^Number of isolates in which respectively the gene, mRNA or protein was detected are shown. - indicates that the gene, mRNA or protein was not detected in any isolate.

*ND*: not determined due to low signal intensities with coefficients of variation larger than 25% between duplicate experiments.

**Table S2. *S. aureus* protein-specific IgG levels in controls and patient groups.**

| Antigen | Functional class | Mean IgG level control patients (± SD; N=20) | Mean IgG level bacteremia patients (± SD; N=10)^1^ | Mean IgG level osteomyelitis patients (± SD; N=10)^1^ | P value ANOVA^2^ | P value Post-hoc analysis^3^ |
| --- | --- | --- | --- | --- | --- | --- |
| Alpha toxin | toxin | 8895 (± 4419) | 11610,9 (± 5117) | 14884,3 (±3749) | 0,037 | 0,011 (osteomyelitis) |
| CHIPS | immmune modulator | 6578 (±3181) | 7788 (±2854) | 8670 (±3003) | 0,105 |  |
| ClfA | surface protein | 2375 (±2580) | 2935 (±2639) | 2601 (±1758) | 0,753 |  |
| ClfB | surface protein | 1736 (±1706) | 2244 (±1893) | 2775 (±1751) | 0,321 |  |
| Efb | immmune modulator | ND | ND | ND | ND |  |
| EsxB | housekeeping | ND | ND | ND | ND |  |
| ETA | toxin | 893 (±1425) | 1238 (±1946) | 2874 (±4178) | 0,026 | 0,007 (osteomyelitis) |
| ETB | toxin | 1194 (±1579) | 575 (±787) | 1210 (±2107) | 0,401 |  |
| FlipR | immmune modulator | 1864 (±1569) | 4490 (±3485) | 3656 (±2143) | 0,007 | 0,019 (osteomyelitis + bacteremia) |
| FnbA | surface protein | 332 (±358) | 596 (±577) | 686 (±1248) | 0,079 |  |
| FnbB | surface protein | 599 (±625) | 587 (±840) | 439 (±275) | 0,656 |  |
| Glucosaminidase | housekeeping | 5273 (±2827) | 86780 (±3612) | 8377 (±3882) | 0,005 | 0,019 (osteomyelitis + bacteremia) |
| HlgB | toxin | 6328 (±4290) | 10838 (±2924) | 10917 (±3627) | 0,002 | 0,007 (osteomyelitis + bacteremia) |
| IsaA | housekeeping | 6102 (±3676) | 9373 (±4930) | 7860 (±5526) | 0,240 |  |
| IsdA | surface protein | 3722 (±4532) | 6534 (±3993) | 5016 (±2756) | 0,006 | 0,023 (osteomyelitis + bacteremia) |
| IsdH | surface protein | 825 (±867) | 2579 (±3130) | 2732 (±4407) | 0,034 | 0,011 (bacteremia) |
| Lipase | housekeeping/ toxin | 4072 (±2550) | 6557 (±4197) | 7155 (±4412) | 0,251 |  |
| LukD | toxin | 6311 (±3988) | 9512 (±3514) | 9369 (±3581) | 0,024 | 0,044 (osteomyelitis + bacteremia) |
| LukE | toxin | 8859 (±4327) | 11743 (±3621) | 11510 (±3494) | 0,087 |  |
| LukF | toxin | 1105 (±880) | 1851 (±772) | 2025 (±917) | 0,001 | 0,005 (osteomyelitis + bacteremia) |
| LukS | toxin | 8382 (±4659) | 10988 (±4031) | 10518 (±3915) | 0,127 |  |
| LytM | housekeeping | 950 (±1706) | 1171 (±1500) | 900 (±1295) | 0,971 |  |
| Nuc | housekeeping/ toxin | 2412 (±2697) | 4430 (±3612) | 3215 (±1882) | 0,132 |  |
| PrsA | housekeeping | ND | ND | ND | ND |  |
| SACOL0486 | housekeeping | ND | ND | ND | ND |  |
| SACOL0688 | housekeeping | 839 (±650) | 3849 (±4290) | 2572 (±2432) | 0,001 | 0,005 (osteomyelitis + bacteremia) |
| SasG | surface protein | 391 (±673) | 270 (±293) | 299 (±269) | 0,786 |  |
| SCIN | immmune modulator | 3665 (±3322) | 7939 (±3805) | 7545 (±3782) | <0,000 | 0,002 (osteomyelitis + bacteremia) |
| SdrD | surface protein | 668 (±480) | 1306 (±1620) | 992 (±1158) | 0,437 |  |
| SdrE | surface protein | 293 (±298) | 399 (±385) | 651 (±441) | 0,026 | 0,007 (osteomyelitis) |
| SEA | toxin | 3219 (±3300) | 3199 (±3135) | 3669 (±2842) | 0,571 |  |
| SEB | toxin | 2996 (±3016) | 3969 (±3906) | 4613 (±3714) | 0,244 |  |
| SEC | toxin | 9164 (±4334) | 8098 (±4407) | 9157 (±3739) | 0,633 |  |
| SED | toxin | 866 (±909) | 1322 (±1338) | 1794 (±2249) | 0,231 |  |
| SEE | toxin | 1344 (±2024) | 1190 (±1493) | 1266 (±1428) | 0,749 |  |
| SEG | toxin | 745 (±966) | 907 (±1913) | 958 (±1136) | 0,651 |  |
| SEH | toxin | 2044 (±1920) | 1584 (±1667) | 3079 (±2799) | 0,219 |  |
| SEI | toxin | 664 (±539) | 1268 (±1661) | 2009 (±1561) | 0,157 |  |
| SEM | toxin | 526 (±542) | 1155 (±1595) | 1322 (±966) | 0,028 | 0,011 (osteomyelitis) |
| SEN | toxin | 677 (±910) | 665 (±995) | 703 (±1141) | 0,967 |  |
| SEO | toxin | 120 (±65) | 223 (±317) | 361 (±416) | 0,060 |  |
| SEQ | toxin | 1120 (±1599) | 1900 (±3039) | 3274 (±3907) | 0,372 |  |
| SER | toxin | 1265 (±1985) | 834 (±1187) | 1501 (±3436) | 0,869 |  |
| SSL1 | immmune modulator | 2794 (±2281) | 4848 (±3456) | 4887 (±3477) | 0,117 |  |
| SSL3 | immmune modulator | 4679 (±3068) | 8186 (±4746) | 6955 (±3673) | 0,011 | 0,042 (osteomyelitis + bacteremia) |
| SSL5 | immmune modulator | 1929 (±1307) | 4675 (±3315) | 3932 (±2827) | 0,001 | 0,014 (osteomyelitis + bacteremia) |
| SSL9 | immmune modulator | 6835 (±3575) | 9240 (±3735) | 8892 (±4048) | 0,115 |  |
| SSL10 | immmune modulator | 3051 (±4038) | 5032 (±3893) | 5425 (±4038) | 0,175 |  |
| SSL11 | immmune modulator | 1087 (±1540) | 1332 (±1297) | 1793 (±2149) | 0,425 |  |
| TSST1 | toxin | 8217 (±6646) | 7865 (±3942) | 9110 (±4057) | 0,545 |  |

^1^Only the peak IgG levels of patients were included for comparison.

^2^P value of ANOVA test comparing all three patient groups. P values < 0.05 were considered as significant.

^3^P value of post-hoc tests with LSD are shown (patient group(s) with significantly higher mean IgG level(s) compared to controls are indicated).

ND: not determined due to low signal intensities with coefficients of variation larger than 25% between duplicate experiments.
