# Supplementary material for: Combining in vitro protein detection and in vivo antibody detection identifies potential vaccine targets against Staphylococcus aureus during osteomyelitis
Source: Med Microbiol Immunol. 2016 Sep 14;206(1):11–22. doi: 10.1007/s00430-016-0476-8 (PMC5263195; doi:10.1007/s00430-016-0476-8)
Supplement: Supplementary file 3 — Supplementary material 3 (DOC 209 kb) [file 430_2016_476_MOESM3_ESM.doc]

**Online Resource 3. Supporting Figures.**

**Fig. S1**

**
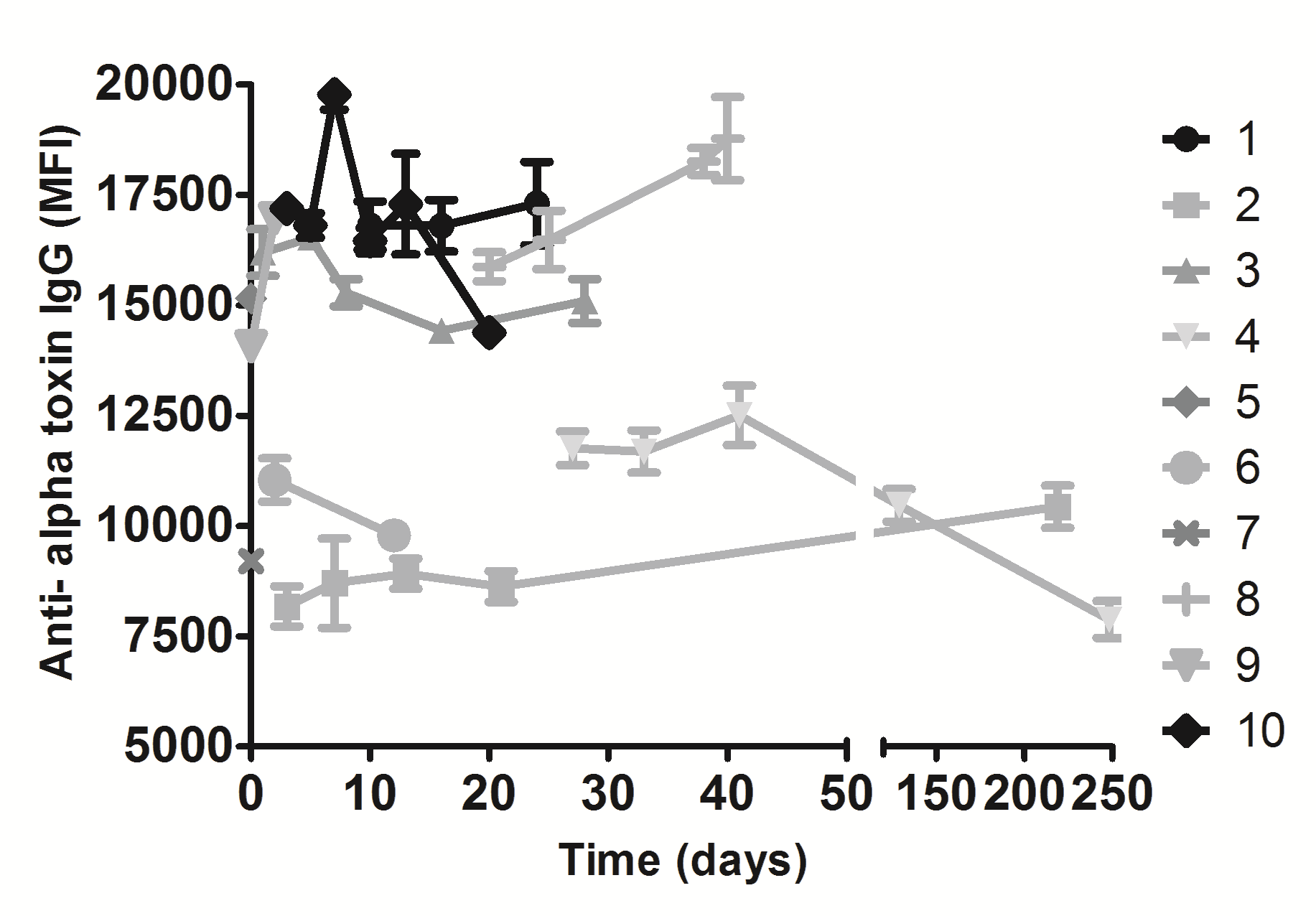
**

**Fig. S1. Height and course of anti-alpha toxin IgG levels in 10 patients diagnosed with chronic osteomyelitis.** Time point 0 was arbitrarily defined as the date of the first deep bone culture during the study period. Each data point represents the average of duplicate measurements expressed as the mean fluorescence intensity (MFI).

**Fig. S2**

**
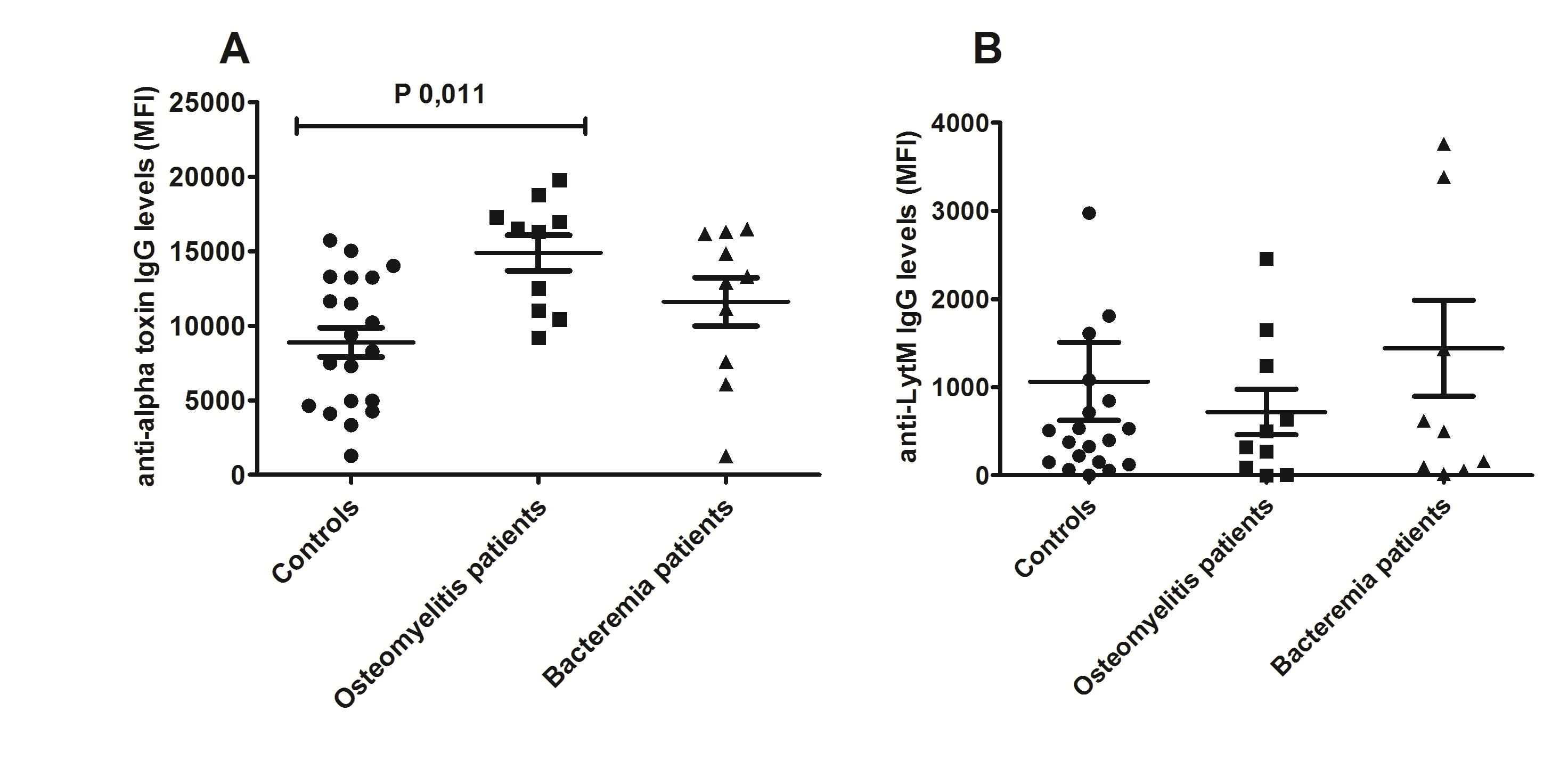
**

**Fig. S2. Comparison of IgG levels between patients and non-infected controls.** Peak IgG levels of 10 patients suffering from either chronic osteomyelitis or bacteremia and 20 non-infected age-matched controls are compared for alpha toxin (**A**) and LytM (**B**). Average IgG levels against alpha toxin differed significantly between controls and osteomyelitis patients (P value indicated). IgG levels against LytM did not significantly differ between groups. The median value and interquartile range are represented by lines.

**Fig. S3**

**
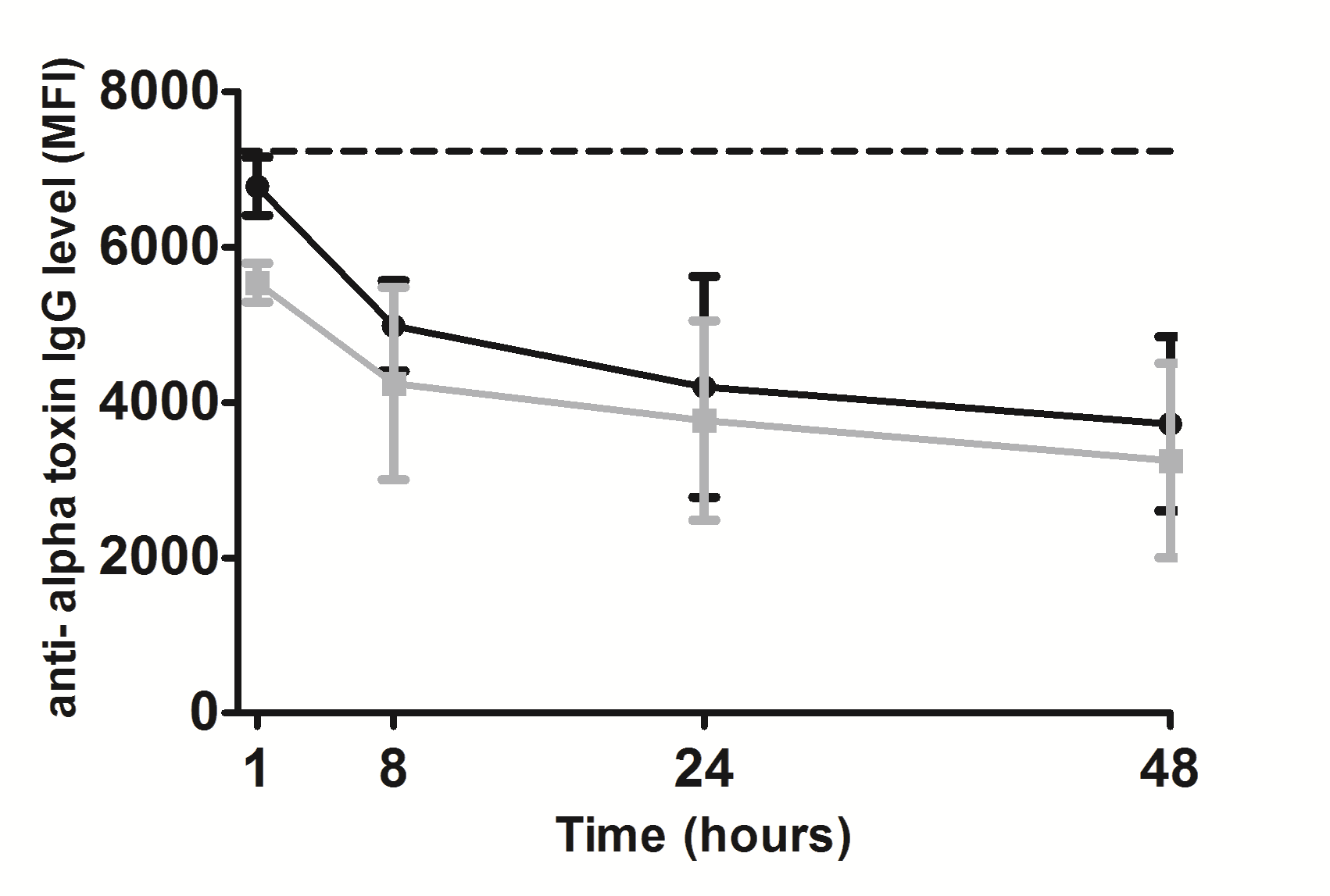
**

**Fig. S3. Detection of alpha toxin in 24hrs-biofilms of 10 strains and surrounding medium.** The mean remaining non-bound IgG against alpha toxin was separately measured after incubation of PHG with biofilms of 10 strains on PS (dark circles) and after incubation with the IMDM culture medium covering the biofilms (light squares). Error bars indicate standard deviation and the dashed horizontal line indicates the average MFI of sterile controls. Similar results were obtained for the other secreted antigens (data not shown).
